# *de novo* Design and Synthesis of *Candida antarctica* Lipase B Gene and α-Factor Leads to High-Level Expression in *Pichia pastoris*

**DOI:** 10.1371/journal.pone.0053939

**Published:** 2013-01-10

**Authors:** Jiang-Ke Yang, Li-Ying Liu, Jiang-Hong Dai, Qin Li

**Affiliations:** 1 College of Biological and Pharmaceutical Engineering, Wuhan Polytechnic University, Wuhan, Hubei, China; 2 School of Life Science, Huazhong University of Science and Technology, Wuhan, Hubei, China; Louisiana State University, United States of America

## Abstract

*Candida antarctica* lipase B (CALB) is one of the most widely used and studied enzymes in the world. In order to achieve the high-level expression of CALB in *Pichia*, we optimized the codons of CALB gene and α-factor by using a *de novo* design and synthesis strategy. Through comparative analysis of a series of recombinants with different expression components, we found that the methanol-inducible expression recombinant carrying the codon-optimized α-factor and mature CALB gene (pPIC9KαM-CalBM) has the highest lipase production capacity. After fermentation parameters optimization, the lipase activity and protein content of the recombinant pPIC9KαM-CalBM reached 6,100 U/mL and 3.0 g/L, respectively, in a 5-L fermentor. We believe this strategy could be of special interest due to its capacity to improve the expression level of target gene, and the *Pichia* transformants carrying the codon-optimized gene had great potential for the industrial-scale production of CALB lipase.

## Introduction

*Candida antarctica* lipase B (CALB) is firstly purified from the secretion components of *C. antarctica*, which is a yeast isolated from the sediment in Lake Vanda, Victoria Land in Antarctica [1]. The use of CALB in biocatalysis has steadily increased in the recent years, and now it is one of the most widely used and studied enzymes. The natural reaction of CALB is ester hydrolysis. This hydorlysis process reacts through a connective Ser intermediate proton and OH^−^ transfer. OH^−^ supplied by water molecule can attack the serine hydroxyl group which covalent binding with the substrate carbonyl carbon atom to form the carboxylic acid; Simultaneously, the proton is transferred by histidine from the water molecule to the serine anion oxygen to form serine −OH, and released the free carboxyl compounds. In non-aqueous phase, CALB can use other substrates as OH^−^ supplier (nucleophile) to mediates a series of biochemical reactions such as esterification and transesterification [2]. CALB is an efficient biocatalyzer in non-aqueous environment. It can be used in a broad range of fields, and possesses excellent catalytic performance than any other lipases in terms of biodiesel production [3], [4], polymer synthesis [5], chiral resolution [6] and pharmaceuticals preparation [7].

Since it was firstly cloned from *C. antarctica* LF058, CALB has been successfully expressed in a series of hosts, such as *Escherichia coli*, *Aspergillus oryzae* [8]–[10] and *Pichia pastoris* [11]–[12]. *E. coli* is widely used for gene expression. However, the product of CALB gene in *E. coli* is insoluble inclusion body in most cases, and requires a complicated refolding process to get the active form [11], [13]. *P. pastoris* is now broadly used as an expression system for the prodution of recombinant heterologous lipase due to many advantages such as high growth rate, efficient expression capacity, simple nutrient utilization and also suitability for high-density fermentation [14]–[18].

The heterologous gene expression can be significantly affected by the codon usage frequency. Like most organisms, *Pichia* displays a non-random pattern of synonymous codon usage and shows general bias towards a subset of codons, leading to an affected heterogenous expression efficiency in *Pichia*. In order to improve the expression level, codon optimization has been established as an efficient measure by replacing rarely used codons with frequently used ones [19]–[23]. Moreover, factors, such as the complexity of mRNA secondary structures, A/T rich region leading to the expression pre-termination and the degree of sequence identity to homologs, can also affect the expression level and must be simultaneously considered. With the in-depth understanding of gene expression and development of bioinformatics tools [24], *in silico* designing and *in vitro* gene synthesis strategy become more and more popular in molecular rebuilding [19], [20], [22].

In order to realize the high-level expression of CALB gene in *P. pastoris*, we optimized the codons of both CALB gene and α-factor signal peptide using a *de novo* design and synthesis strategy addressing above expression-related issues. Moreover, in order to obtain the high efficient expression recombinants, we also investigated the factors such as the constitutive or inducible expression, signal peptide type, pre-sequence of CALB and the fermentation parameters for enzyme production.

## Materials and Methods

### de novo CALB Gene and α-factor Design and Synthesis

**Figure 1:**
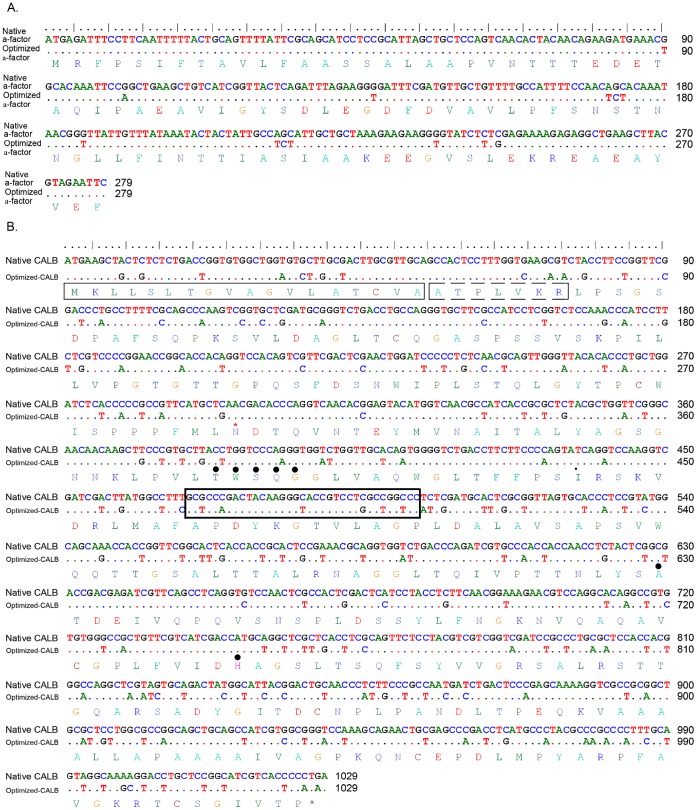
Sequence comparison between the native and codon-optimized genes. (A). α-factor; (B). CALB gene. Dots represent the same nucleotides between the native and codon-optimized genes. Solid line box and dash line box indicate the signal peptide and pre-sequence of CALB, respectively, and * indicates the possible glycosylation site. • indicate the catalytic triad Ser_130_–Asp_210_–His_249_ and the conserved penta-peptide motif TWS_130_QG. Bold solid line box indicate the link sequence of F1 and F2 fragments for OE-PCR.

Codons of CALB gene were optimized according to the native nucleic acid and amino acid sequences of CALB of *C. antarctica* LF 058 (GenBank: Z30645; P41365). The usage frequency of codons in *Pichia* genome was determined by referring to the codon usage database (http://www.kazusa.or.jp/codon/), and the codon usage frequency in native and codon-optimized CALB genes was analyzed online by graphical codon usage analyser software 2.0 (http://gcua.schoedl.de/). The Less frequently used codons in *Pichia* were replaced with the frequently used ones by DNA2.0 software (http://www.dna20.com). In order to optimize the α-factor signal peptide used in expression vector pPIC9K, eight least frequently used codons were simply replaced with the most frequently used ones (Fig. 1, Fig. S1 and Fig. S2). The full-length sequence of CALB gene was divided into two fragments (F1 and F2; F1M and F2M) with approximately a 20-bp overlap at each end. The oligonucleotides of 20–50 bp to assemble the F1, F2, F1M, F2M and α-factor fragments were designed by Gene2Oliga software [24] to make the thermodynamic properties of each oligonucleotide consistent, and synthesized by Sangon Ltd. China. Table S1 to S5 list the oligonucleotides used to synthesize the native and codon-optimized CALB genes and α-factor in our study.

### Full-length CALB Gene and α-factor Signal Peptide Assembly

The native and codon-optimized CALB genes were synthesized by a two-step gene synthesis strategy that combined an assembly PCR and an overlap extension PCR (AOE) [25]. In the first step, the oligonucleotides were separately assembled into fragments F1 and F2 by assembly PCR (A-PCR). This amplification was carried out in a 50-µL reaction system containing 200 µM of dNTPs, 0.1 mM of each oligonucleotide, 1.5 mM MgCl_2_ and 1 U of *Pfu* Turbo DNA polymerase (Stratagene, La Jolla, CA). The A-PCR were performed using the following cycle conditions: pre-denaturation at 94°C for 2 min; 30 cycles at a melting temperature of 94°C for 30 sec, an annealing temperature of 52°C for 30 sec and an extension temperature of 72°C for 1 min; Finally, an extra extension step of 72°C for 6 min. The amplicons were used as the templates in another round of PCR using two outer primers. The amplification was performed in a 50-µL reaction system containing 3 µL of A-PCR mixture, 200 mM of dNTPs, 1 mM of each primer, 1.25 mM of MgCl_2_ and 1 U of *Pfu* DNA polymerase.

PCR products of F1 and F2 fragments were 1∶50 diluted and then subjected to the overlap extension PCR (OE-PCR) with the outer primers containing the restriction digestion sites (Table S6 and S7). A 50-µL reaction contained 200 µM of dNTPs, 0.1 mM of each primer and 1 U *Pfu* DNA polymerase. Briefly, following a pre-denaturation step at 94°C for 2 min, amplification was carried out with 30 cycles at a melting temperature of 94°C for 30 sec, an annealing temperature of 52°C for 30 sec and an extension temperature of 72°C for 1 min. Finally, an extra extension step of 72°C for 5 min was followed. Subsequently, the PCR products were subjected to dA tailing and cloned into pMD18-T vector (Takara, Dalian, China).Three positive clones were selected and the insertion fragments were sequenced to confirm the synthesis accuracy. The α-factor signal peptide (279 bp) was synthesized by a single-step A-PCR using the same procedure mentioned above.

### Plasmid Construction, Transformation and Transformant Selection

Methanol-inducible expression vector pPIC9K, pPIC3.5K and constitutive expression vector pGAPZα were used for the cloning and expression of CALB in *P. pastoris*. Both plasmid pPIC9K and pGAPZα contained a α-factor signal peptide from *Saccharomyces cerevisiae* for directing the protein to the secretary pathway, whereas it was missing in the plasmid pPIC3.5K. Codon-optimized α-factor signal sequence (αM) was introduced into pPIC9K by simply replace the native α-factor signal sequence through restriction sites *BamH* I and *EcoR* I to generate the plasmid pPIC9KαM. In order to make the CALB co-expressed with α-factor, two restriction sites *EcoR* I and *Not* I were introduced into the PCR products of native (CalB) and codon-optimized (CalBM) CALB genes, and then they were inserted into pPIC9K, pPIC9KαM, pPIC3.5K and pGAPZα to generate plasmids pPIC9K-CalBP, pPIC9K-CalB, pPIC9K-CalBM, pPIC9KαM-CalB, pPIC9KαM-CalBM, pGAPZα-CalB and pGAPZα-CalBM, respectively. CALB gene containing native signal peptide and pre-sequence (CalBSP) was cloned into pPIC3.5K by *BamH* I and *Not* I sites to generate plasmid pPIC3.5K-CalBSP.

About 5 µg of plasmid DNA linearized by *Sac* I was mixed with 80 µL of competent cells, and then it was transformed into *Pichia* cells by electroporation conducted on Gene Pulser (Bio-rad, Richmond, CA) according to the manufacturer’s instruction for *S. cerevisiae* to generate stable *P. pastoris* transformants via homologous recombination at the AOX1 locus between the transforming DNA and regions of homology within the *Pichia* genome. Positive clones were initially selected on MD medium (1.34% yeast nitrogen base, 4×10^−5^% biotin, 2% dextrose) plates and then confirmed by colony PCR.

### Fermentation

A single colony of transformants was selected and inoculated into 50 mL BMGY (1% yeast extract, 2% peptone, 100 mM potassium phosphate buffer with pH 6.0, 1.34% yeast nitrogen base, 4×10^−5^% biotin, 1% glycerol) medium in flask, and cultured at 28°C in a shaking incubator (250 rpm) until an OD_600_ of 3.0 was obtained. The cells then were harvested and transferred into 50 mL BMMY (1% yeast extract, 2% peptone, 100 mM potassium phosphate buffer pH 6.0, 1.34% yeast nitrogen base, 4×10^−5^% biotin, and 0.5% methanol) medium for the methanol-inducible expression for another 4 days. Methanol at a final concentration of 0.5% was added every 24 h to induce the lipase expression, and the lipase activity was checked at intervals. A total of eight types of constructed transformants were checked by flask fermentation, and for each transformant three replicates were conducted.

The transformant with the highest lipase activity in flask was selected for the high density fermentation in a 5-L Biostat fermentor (B.Braun Biotech International, Melsungen, Germany). A fed-batch fermentation process was performed according to the model protocol described by the Invitrogen (http://toolszh.invitrogen.com/content/sfs/manuals/ pich_man.pdf). The fermentation basal salts (BSM) (H_2_PO_4_ 26.7 mL, CaSO_4_ 0.93 g, K_2_SO_4_ 18.2 g, MgSO_4_•7H_2_O 14.9 g, KOH 4.13 g, glycerol 40.0 g, per liter) were used for yeast cell culture, and the parameters were monitored and controlled throughout the whole fermentation process. Briefly, the fermentation parameters were maintained as follows: temperature (27.0°C), dissolved oxygen (DO,>30%), pH (6.0), agitation (rpm, 550–650) and aeration (0.1–1.0 vvm). For the inducible expression of lipase, methanol was added into the broth at a final concentration of 0.5%. The time point for methanol induction was 30 h, and the methanol was fed every 12 h with 0.5 mL/min speed. The whole fermentation time was 140 h and the methanol-induction time was 110 h. Samples were collected at intervals, and the fresh cell weight, lipase activity and protein content in broth were analyzed. Cell growth was monitored at various time points by determining the fresh cell weight (g/L). Purification of the lipase was conducted according to the description of Yang et al. [26], and the protein content was determined by the Bradford method [27].

### Lipase Activity and Protein Content Assays

To qualitatively analyze the lipase activity, the yeast transformants were inoculated onto the GMMY agar plate (containing 0.5% tributyrin), and the halo diameter around the colonies was measured. Lipase activity was determined at pH 7.5 by free butyric acid titration using 50 mM NaOH. after incubated in a thermostated vessel for 10 min. The assay mixture consisted of 5 mL Tris-HCl buffer (50 mM), 50 mM NaCl, 4 mL emulsified tributyrin and 1 mL diluted enzyme solution. One unit (U) of the activity was defined as the amount of enzyme liberating 1 micromole of butyric acid per min at 45°C.

## Results and Discussion

### de novo Gene Design and Synthesis

*Pichia pastoris,* an easy and simple system suitable for high density fermentation, has been widely used to produce recombinant heterologous proteins, including a series of lipases from different organisms [14]–[18]. However, due to the discrepancy of codon usages between the *Pichia* and original hosts, the expression levels of these lipases hardly reach their optima. We compared the codon usages for *C. antartica* and *P. pastoris* and identified significant differences (Fig. 1). For example, codons for amino acids Leu (CTC), Ala (GCG), Ser (TCG) and Pro (CCG) in *C. antarctica* were very infrequently used in *P. pastoris* genome (Table S8, Fig. S2). With the in-depth knowledge of gene expression and reduce of cost on oligonucleotides synthesis, *de novo* desiging and whole gene synthesis technology have gradually been used to transform the coding sequence to be more in line with the host cell codon. Previous reports have also demonstrated that it is a simple and fast way to achieve effective expression of foreign gene [19], [28].

In order to achieve a high-level expression in *P. pastoris*, we replaced the less frequently used codons of CALB gene with those more frequently used (Table S8, Fig. 1 and Fig. S2). During the gene designing process, the following five factors affecting the expression efficiency of CALB gene were considered: 1) The least frequently used codons which will be the bottleneck of gene expression was directly replaced by the highest or second highest frequently used codons; 2) In order to make nucleotides A, T, G and C evenly dispersed in the synthesized gene, degenerate codons containing both AT and GC bases were selected when the differences between the codon frequencies were not significant; 3) the GC content of synthesized gene was kept within 45–55%; 4) to prevent the exhaustion of frequently used tRNA, the codons of some amino acids, such as Leu, Thr, Ala and Gly, were replaced by the second or third high-frequency codons. For example, although the highest frequency codon for Leu is TTG (31.9), the usage frequency for other two degenerate codons CTT (16.1) and CTG (15.5) was still acceptable. When we met the amino acid sequence block such as FML_98_N and YL_229_FN (Fig. 1), if we always select the highest-frequency codon for each amino acid (Table S8), the nucleotide sequences will become 5′-TTTATGTTGAAC-3′ and 5′-TACTTGTTTAAC-3′, respectively. So in order to make the four nucleotides dispersing in the sequence evenly and also to make the GC content within 45%–55%, the second high-frequency codon for Phe (TTC, 18.9) and the third high-frequency codon for Leu (CTG, 15.5) were selected and the nucleotide sequencs of these blocks becoming 5′-TTCATGCTGAAC-3′ and 5′-TACCTGTTCAAC-3′, respectively (Fig. 1). 5) Since the expression level of glycosylation-site-free CALB is equal to that with the glycosylation site [11], therefore, the glycosylation site (74Asn) of CALB was retained (Fig. 1). Comprehensively, about 170 rarely used codons were optimized (Fig. 1B). The GC content of gene was decreased from 61.89% to 53.99%. Moreover, we also optimied the codon of α-factor by simply replacing nine rarely used codons with those frequently used ones (Fig. 1A). After codon optimization, the minimal free energy (MFE) was increased from −386.5 kcal/mol to −269.56 kcal/mol, indicating the decreased complexity of the secondary structure of mRNA (Fig. S3).

### Assembly of α-factor and CALB Gene

**Figure 2:**
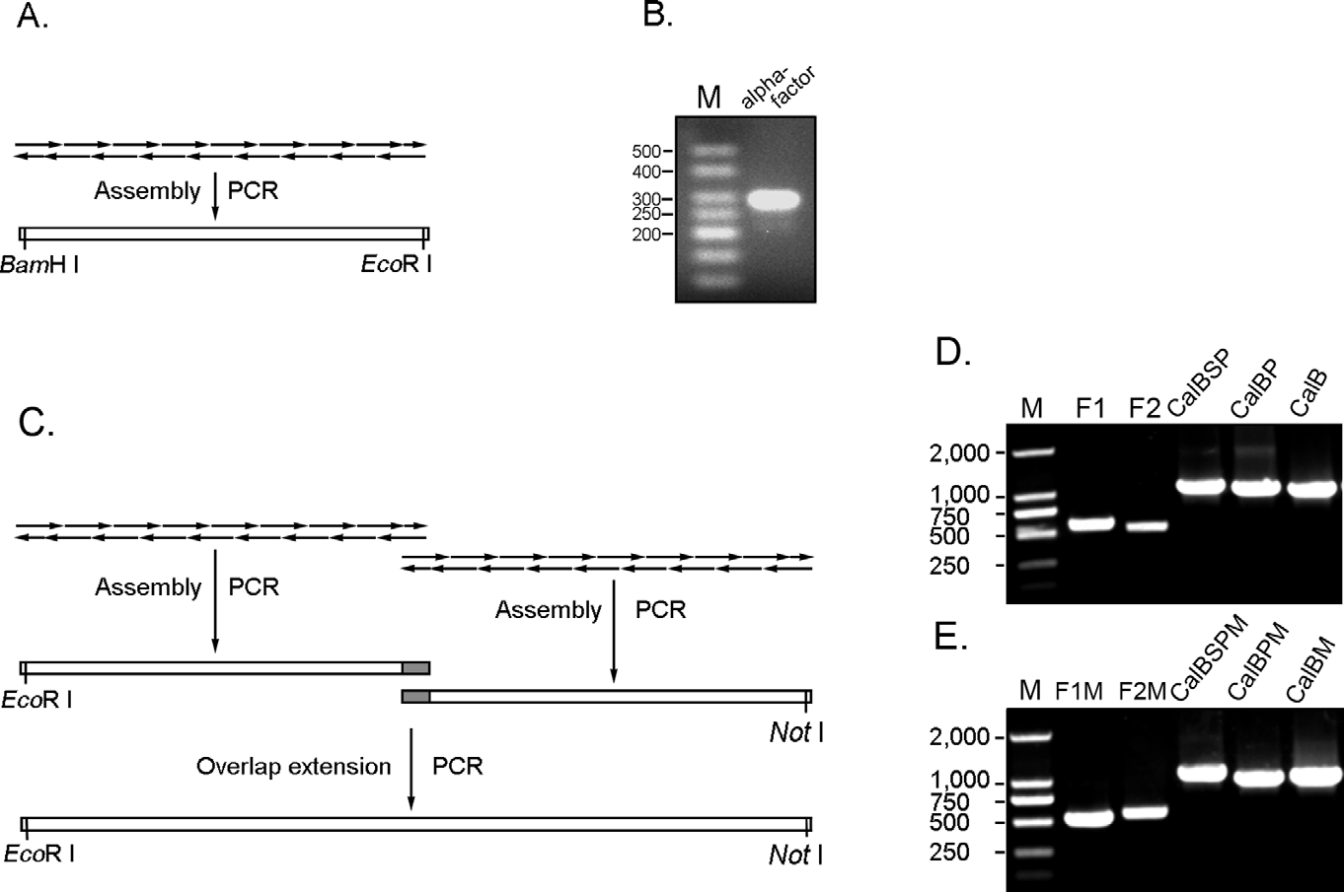
*in vitro* synthesis of α-factor, native CALB and codon-optimized CALB genes. A single-step strategy (A-PCR) was conducted to synthesize the codon-optimized α-factor (A and B), and a two-step strategy combining A-PCR and OE-PCR (C) was conducted to synthesize the native CALB (D) and codon-optimized CALB (E) genes. In order to synthesize the native CALB, the oligonucleotides were firstly assembled into F1 (541 bp) and F2 (510 bp), and then they were assembled into the genes with native signal peptide (CalBSP), native pre-sequence (CalBP) and mature CALB (CalB) with different primer pairs at OE-PCR step (D). In order to synthesize the codon-optimized CALB, the oligonucleotides were firstly assembled into F1M (510 bp) and F2M (553 bp), and then they were assembled into genes with signal peptide (CalBSPM), pre-sequence (CalBPM) and mature CALB (CalBM) with different primer pairs at OE-PCR step (E).

In this study, we assembled the α-factor signal peptide using a single-step A-PCR procedure (Fig. 2A and 2B). Since mis-priming frequently occurs as the number of primers increases, long DNA sequences (>0.5 kb) are difficult to synthesize by a single-step procedure. Serious mismatches between oligonucleotides can prematurely terminate the reaction and form the premature DNA products. To overcome these problems, two-step gene synthesis methods employing a PCR step (dual asymmetric PCR or A-PCR) to produce several fragments and then assembling them into a long DNA sequence by OE-PCR has been developed for long DNA sequence synthesis [28]−[30]. In this study, we synthesized the native and codon-optimized CALB genes with a two-step strategy combining A-PCR and OE-PCR procedure (Fig. 2C to 2E). In the first step, we conducted the A-PCR to assemble the synthesized oligonucleotides covering both strands of DNA molecule into two fragments (F1 and F2, F1M and F2M). This step was similar to the general method of single-step A-PCR gene synthesis [31]. In the second step, we conducted an OE-PCR to assemble two fragments into the full-length genes (Fig. 2C, 2D and 2E). In order to synthesize genes with different components, we used the different prime pairs (Table S6 and S7) to amplify the genes with the native or codon-optimized signal peptide, pre-sequence and mature CALB genes in the OE-PCR step (Fig. 2D and 2E).

### Expression in P. pastoris

**Figure 3:**
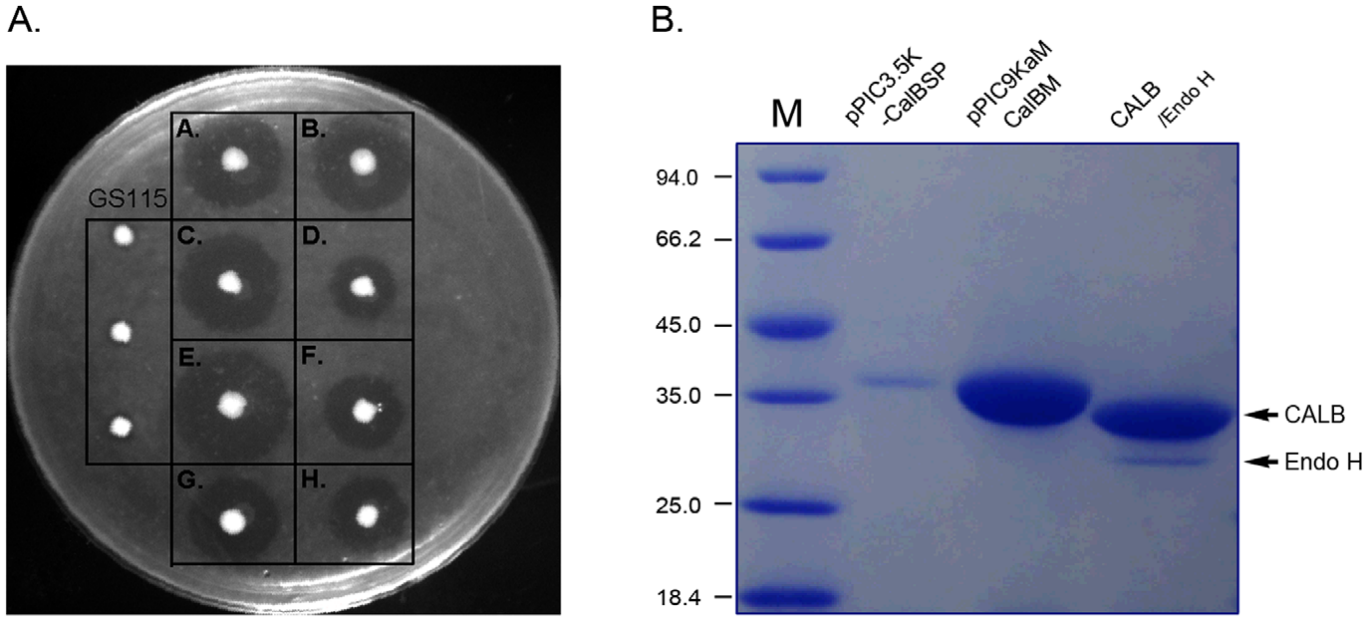
Lipase activity of the recombinants. (A). The phenotypes of the recombinants on the tributyrin-MS plates; (B). The expression products of the recombinants. In Fig. 3A, A: pGAPZα-CalBM, B: pPIC9KαM-CalB; C: pPIC9K-CalBM, D: pPIC3.5K-CalBSP, E: pPIC9KαM-CalBM, F: pPIC9K-CalBP, G: pPIC9K-CalB, H: pGAPZα-CalB. In Fig. 3B, a total of 30 µL fermentation broth of pPIC3.5K-CalBSP and pPIC9KαM-CalBM were added into the wells, respectively. The purified CALB was deglycosylation by Endo H and then directly loaded into the well.

The premature CALB contains three parts, N-terminal signal peptide, pre-sequence and mature enzyme (Fig. 1B). In order to obtain a recombinants with the highest expression capacity, the factors including the codon usage frequency, signal peptide, pre-sequence and constitutive or inducible expression were considered. We constructed a series of recombinants and comparatively analyzed their lipase production capacity using tributyrin-MS plates and flask fermentation (Fig. 3A). The lipases were expressed as a glycosylized secreting proteins from both the original and synthesized genes with the size of 37 kDa, and after deglycosylation by Endo H the size becoming 35 kDa (Fig. 3B).

**Figure 4:**
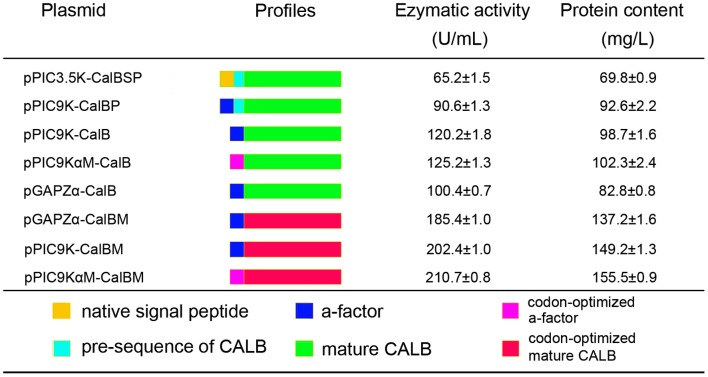
Lipase production capacity of the recombinants checked by flask fermentation.

The secretion capacity of α-factor signal peptide was significantly stronger than that of the original signal peptide. For example, the lipase activity of the recombinants pPIC3.5K-CalBSP and pPIC9K-CalBP were 65.2 U/mL, 69.8 mg/L respectively. Howerer, the pre-sequence can retard the CALB expression as showed by pPIC9K-CALBP and pPIC9K-CALB. The recombinants carrying the codon-optimized α-factor signal peptide and CALB gene (pPIC9KαM-CalBM and pGAPZα-CalBM) demonstrated a much stronger lipase secretion capacity than the transformants with original gene (pPIC9K-CalB, pPIC9KαM-CalB, pGAPZα-CalB). The highest activity was obtained from the methanol-inducible, codon-optimized α-factor and CALB co-expressed recombinant pPIC9KαM-CalBM. After the inducible expression for 96 h, both the lipase activity and protein content in the broth reached their maximal levels of 210.7 U/mL and 155.5 mg/L, respectively. In contrast, recombinants (pPIC9K-CalB) carrying the original gene had only 120.2 U/mL and 98.7 mg/L, respectively (Fig. 4).

Codon optimization has been established as an efficient measure to overcome the bias on codon usage frequency and significantly improve the expression of foreign gene in *Pichia* [19]−[21]. In this study, the lipase activity and protein content difference between constructed plasmid pairs, pPIC9K-CalB/pPIC9K-CalBM, pPIC9KαM-CalB/pPIC9KαM-CalBM, pGAPZα-CalB/pGAPZα-CalBM, pPIC9K-CalB/pPIC9KαM-CalB, indicated that the expression level of codon-optimized was about 0.8-fold higher than the native CALB gene, and codon-optimization on α-factor can also effectively improve the expression level of CALB gene in *Pichia*. *S. cerevisiae* originated α-factor was broadly used as a signal peptide for secreted expression of foreign gene in *Pichia* expression system. In this study, we compared secreted expression capacity between α-factor and the native signal peptide of CALB (pPIC3.5K-CalBSP/pPIC9K-CalBSP). Coincided with previous report [32], the capacity of α-factor significantly higher (0.4-fold) than the native signal peptide. Although both inducible- and constitutive-expression [33] of foreign gene can reach a ideal level in *Pichia*, and these was no concrete result to defined which type of expression is better, through the comparative analysis between transformants (pGAPZα-CalB/pPIC9K-CalB, pGAPZα-CalBM/pPIC9K-CalBM) we found that in this study the level of methanol inducible expression of CALB gene will higher than the constitutive expression. Comprehensively, through comparatively analyzed the expression level of the transformants carrying difference expression components, we found the methanol-inducible expression transformants carrying the codon-optimized α-factor and CALB gene (pPIC9KαM-CalBM) has the highest expression capacity among all type of transformants.

### Lipase Production in Fermentor

**Figure 5:**
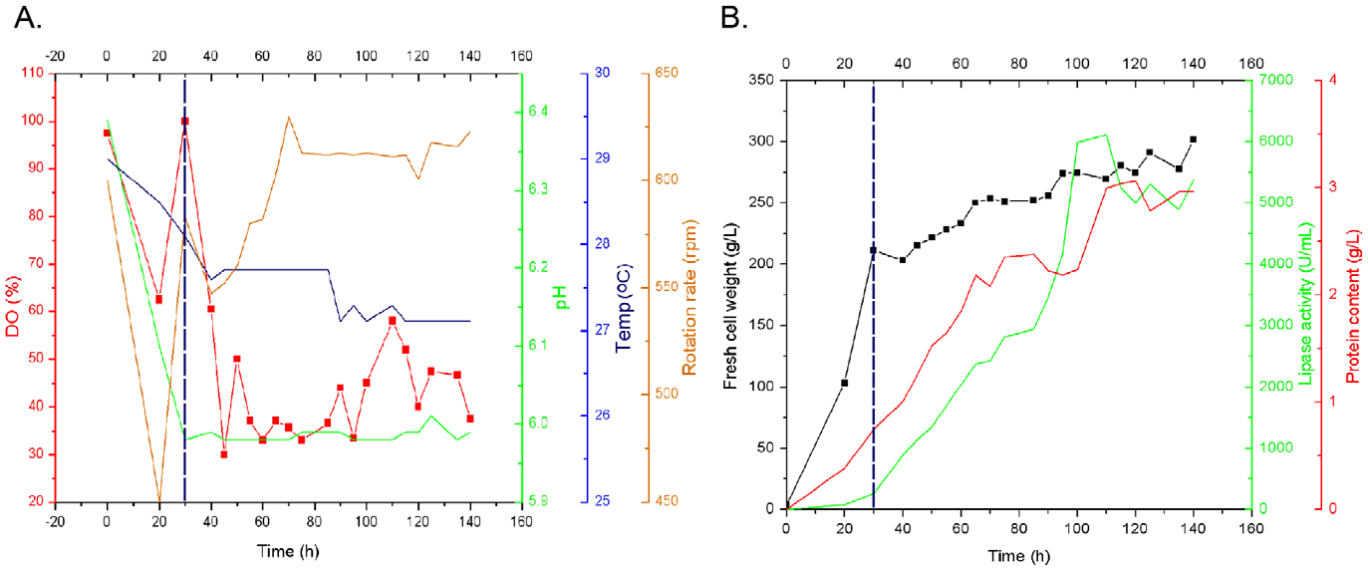
Lipase production of recombinant pPIC9KαM-CalBM in 5-L fermentor. (A). Fermentation condition; (B). Lipase production capacity. The fermentation parameters were maintained as follows: temperature (27.0°C), the pH (6.0) was sustained by ammonia titration, and the dissolved oxygen (DO, >30%) was lined with agitation (rpm, 550–650). For the inducible expression of lipase, methanol was added into the broth at a final concentration of 0.5%. Short dash line indicated the time point for methanol induction.

The lipase production capacity of yeast recombinant could be significantly improved under the batch-induced mode with a tighter control of pH, methanol concentration and aeration conditions. Through fermentation parameters optimization, Jahic et al. [34], [35] had achieved an expression level of 1.5 g/L in fermentor. According to the flask fermentation results (Fig. 5), we selected the yeast transformant with the highest activity (pPIC9KαM-CalBM) for fermentation test in a 5-L fermentor. The parameters such as dissolved oxygen (DO), pH, rotation rate, aeration and temperature were optimized, and the fresh cell weight, lipase activity and protein content in broth were evaluated. In our early work, the key parameters such as temperature and DO (DO were correlated with rotation rate and aeration) were set as Temp = 30.0°C and DO>50% in the whole fermentation stage. This can shorten the glycerol utilization stage into 28 h, and enhance the biomass to 240 g/L. But in methanol-induction stage, the final lipase activity and protein content were as lower as 4,500 U/L and 2.1 g/L, respectively, after methanol induced for 110 h. Fig. 5 shows that under the conditions of pH 6.0, Temp = 27.0°C, DO = 30%–58%, rotation rate = 610 rpm and the late-stage induction with 0.5% methanol for 80 h, the fresh cell weight reached approximately 270.0 g/L, and the lipase activity and protein content in broth were 6,100 U/mL and 3.0 g/L, respectively.

CALB was one of the most widely used and studied enzymes in the world. To improve the expression level of CALB gene and facilitate its biotechnical application, researchers have expressed it in a series of hosts. Early in 2006, Blank and co-workers have realized the functional expression of CALB gene in periplasm of *E. coli* [13]. While the expression level and activity of CALB in *E. coli* were still unsatisfied. The works conducted by Larsen et al. (2008), Jung and Park (2008) showed that the expression level just reached micro-gram level even after codon-optimization [10], [11]. The successful expression of CALB gene happened in *Pichia*. In 2001, Rotticci-Mulder et al. (2001) have fusion-expressed CALB gene with CBD domain of cellulase, and the recombinant proteins (CBD-CALB) were secreted into the culture medium at the level of 25 mg/L [36]. Jahic and co-worker (2002, 2003) have optimized the fermentation condition to improve the biomass (cell dry weight) to the level of 160 g/L by modeling of growth and energy metabolism. While due to the serious proteolysis, the protein content of CBD-CALB was just 1.2 g/L [34]. Later, they improved the expression level to 1.5 g/L in the culture supernatant by combining the optimal pH and the low temperature [35]. In our work, after methanol induction for 80 h, the activity and protein content of codon-optimized CALB reached 6,100 U/mL and 3.0 g/L in the culture supernatant. This mainly due to the codon-optimization of CALB which efficiently improved the codon usage frequency and the expression level, and also the fermentation parameter optimization may also have deduced the degradation and proteolysis of enzyme in the broth.

### Conclusions

Comprehensively, we successfully improved the expression level of CALB in *Pichia* using a *de novo* gene design and synthesis strategy. Comparative analysis of the lipase production capacity of a series of recombinants carrying different components revealed that the methanol-inducible yeast recombinant carrying the codon-optimized α-factor and mature CALB (pPIC9KαM-CalBM) has the highest lipase product capacity. After the methanol-inducible expression for 110 h, the lipase activity reached its maximal level of 6,100 U/mL in a 5-L fermentor. This strategy could be of special interest due to its capacity to improve the expression level in the system of choice and to produce sufficient amounts of biological material for molecular characterization and biotechnological application. In addition, the *Pichia* transformants carrying the codon-optimized gene had great potential for the industrial-scale production of CALB lipase.

## Supporting Information

Figure S1 Codon usage frequency of native (A) and codon-optimized (B) α-factor in *Pichia*.
(TIF)

Figure S2 Codon usage frequency of native (A) and codon-optimized (B) CALB gene in *Pichia*.
(TIF)

Figure S3 **Secondary structure of the first 200 bp of mature CALB mRNA generated by the software RNAfolder.** (A) Native CALB mRNA with the MFE is −70.0 kcal/mol and (B) Codon-optimized CALB mRNA with the MFE is −63.3 kcal/mol.
(TIF)

Table S1 Oligonucloetides for codon optimized alpha-factor synthesis.
(DOC)

Table S2 Oligonucleotides for the synthesis of F1 fragment of native CALB.
(DOC)

Table S3 Oligonucleotides for the synthesis of F2 fragment of native CALB.
(DOC)

Table S4 Oligonucleotides for the synthesis of F1M fragment of codon-optimized CALB.
(DOC)

Table S5 Oligonucleotides for the synthesis of F2M fragment of codon-optimized CALB.
(DOC)

Table S6 Primers used in OE-PCR for amplifying the native CalBSP, CalBP and mature CALB genes.
(DOC)

Table S7 Primers used in OE-PCR for amplifying the codon-optimized CalBSP, CalBP and mature CALB genes.
(DOC)

Table S8 Codon usage frequency of amino acid of original and optimized CALB gene in *Pichia pastoris*.
(DOC)
