# Supplementary figures and images for: de novo Design and Synthesis of Candida antarctica Lipase B Gene and α-Factor Leads to High-Level Expression in Pichia pastoris
Source: PLoS One. 2013 Jan 10;8(1):e53939. doi: 10.1371/journal.pone.0053939 (PMC3542265; doi:10.1371/journal.pone.0053939)

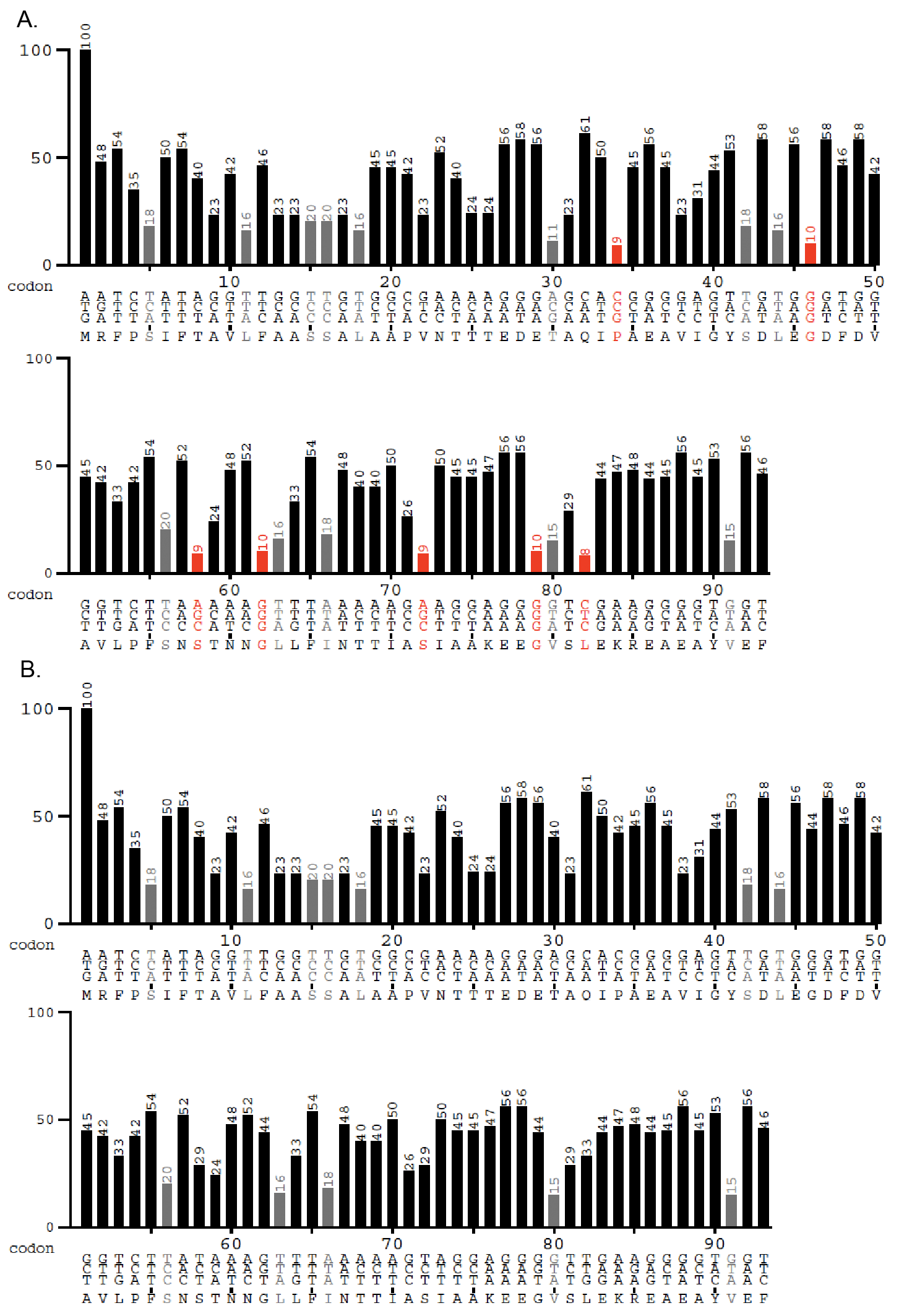

Supplement: Figure S1 — Codon usage frequency of native (A) and codon-optimized (B) α-factor in Pichia. (TIF) [file pone.0053939.s001.tif]

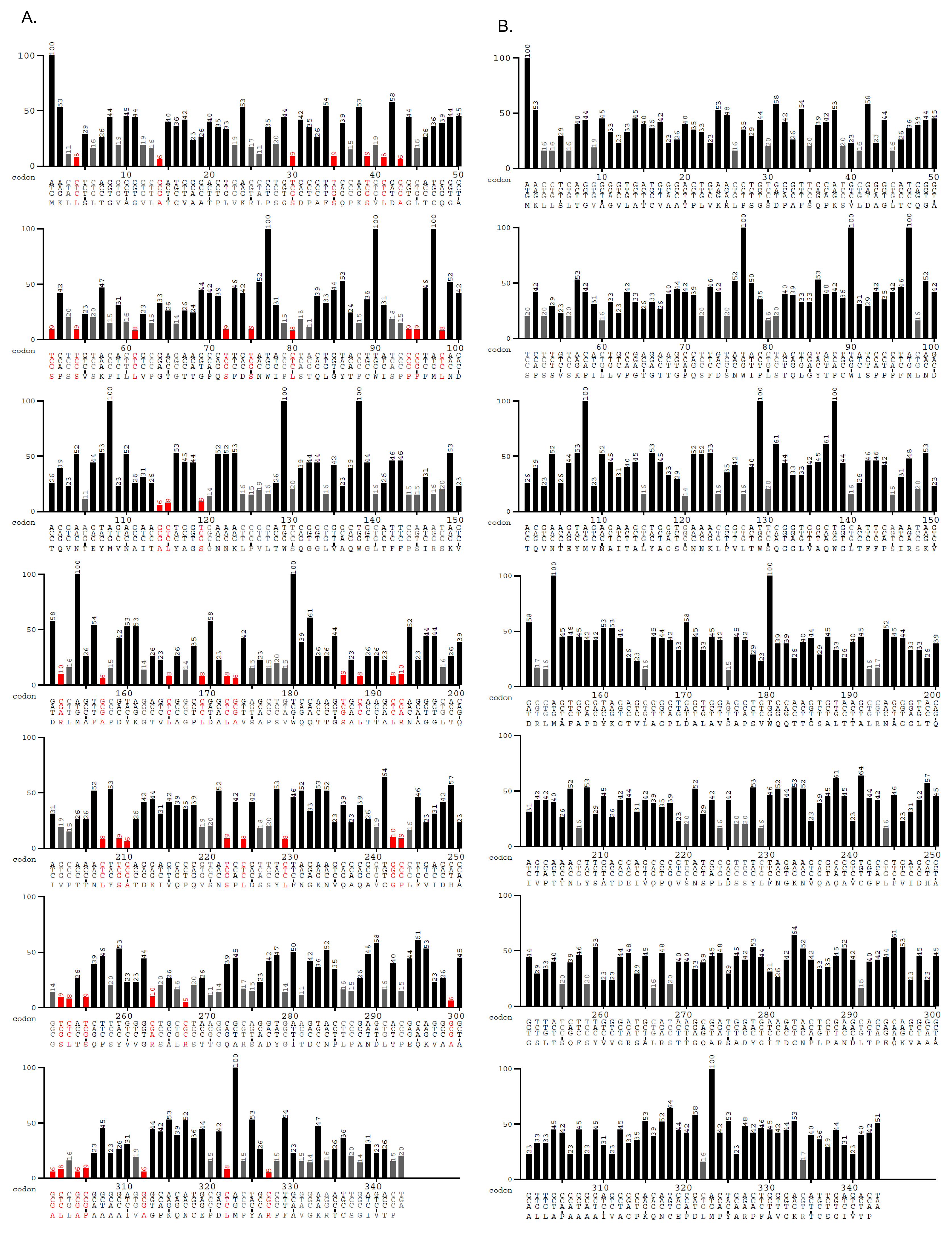

Supplement: Figure S2 — Codon usage frequency of native (A) and codon-optimized (B) CALB gene in Pichia. (TIF) [file pone.0053939.s002.tif]

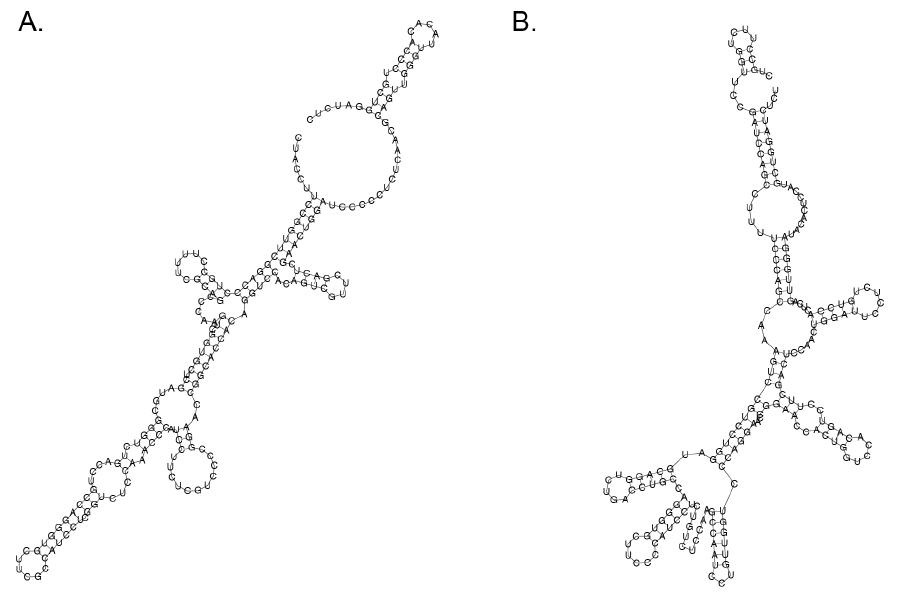

Supplement: Figure S3 — Secondary structure of the first 200 bp of mature CALB mRNA generated by the software RNAfolder. (A) Native CALB mRNA with the MFE is −70.0 kcal/mol and (B) Codon-optimized CALB mRNA with the MFE is −63.3 kcal/mol. (TIF) [file pone.0053939.s003.tif]
