# Supplementary material for: de novo Design and Synthesis of Candida antarctica Lipase B Gene and α-Factor Leads to High-Level Expression in Pichia pastoris
Source: PLoS One. 2013 Jan 10;8(1):e53939. doi: 10.1371/journal.pone.0053939 (PMC3542265; doi:10.1371/journal.pone.0053939)
Supplement: Table S1 — Oligonucloetides for codon optimized alpha-factor synthesis. (DOC) [file pone.0053939.s004.doc]

Table S1 Oligonucloetides for codon optimized alpha-factor synthesis

| ID | Sequence of oligonucleotides (5’-3’) | Number of bps |
| --- | --- | --- |
| R0 | AATCTCATaaagctctaattca | 22 |
| F0 | tgaattagagctttATGAGATTTCCTTCAATTTTTACTGC | 40 |
| R22 | TGCTGCGAATAAAACTGCAGTAAAAATTGAAGGA | 34 |
| F40 | AGTTTTATTCGCAGCATCCTCCGCATTAGCTG | 32 |
| R56 | TGTAGTGTTGACTGGAGCAGCTAATGCGGAGGA | 33 |
| F72 | CTCCAGTCAACACTACAACAGAAGATGAAACTGC | 34 |
| R89 | TTCAGCTGGAATTTGTGCAGTTTCATCTTCTGT | 33 |
| F106 | ACAAATTCCAGCTGAAGCTGTCATCGGTTACT | 32 |
| R122 | GAAATCACCTTCTAAATCTGAGTAACCGATGACAGC | 36 |
| F138 | CAGATTTAGAAGGTGATTTCGATGTTGCTGTTTTGC | 36 |
| R158 | TTTGTAGAGTTGGAAAATGGCAAAACAGCAACATC | 35 |
| F174 | CATTTTCCAACTCTACAAATAACGGTTTATTGTTTATAAATAC | 43 |
| R193 | GCAATAGAGGCAATAGTAGTATTTATAAACAATAAACCGTTA | 42 |
| F217 | TACTATTGCCTCTATTGCTGCTAAAGAAGAAGGTG | 35 |
| R235 | TCTCTTTTCTCCAAAGATACACCTTCTTCTTTAGCA | 36 |
| F252 | TATCTTTGGAGAAAAGAGAGGCTGAAGCTTACGTA | 35 |
| R271 | cttattgagtgaataaaGAATTCTACGTAAGCTTCAGCC | 39 |
| R287 | GAATTCtttattcactcaataag | 23 |
