# Supplementary material for: de novo Design and Synthesis of Candida antarctica Lipase B Gene and α-Factor Leads to High-Level Expression in Pichia pastoris
Source: PLoS One. 2013 Jan 10;8(1):e53939. doi: 10.1371/journal.pone.0053939 (PMC3542265; doi:10.1371/journal.pone.0053939)
Supplement: Table S2 — Oligonucleotides for the synthesis of F1 fragment of native CALB. (DOC) [file pone.0053939.s005.doc]

Table S2 Oligonucleotides for the synthesis of F1 fragment of native CALB

| ID | Sequence of oligonucleotides (5’-3’) | Number of bps |
| --- | --- | --- |
| F1R0 | AGTAGCTTCATGAATTCacacccg | 24 |
| F1F0 | cgggtgtGAATTCATGAAGCTACTCTCTCTGACCGGTGTGGC | 42 |
| F1R24 | CAAGTCGCAAGCACACCAGCCACACCGGTCAGAGAG | 36 |
| F1F42 | TGGTGTGCTTGCGACTTGCGTTGCAGCCACTCCTT | 35 |
| F1R60 | GGAAGGTAGACGCTTCACCAAAGGAGTGGCTGCAACG | 37 |
| F1F77 | TGGTGAAGCGTCTACCTTCCGGTTCGGACCCTGCCT | 36 |
| F1R97 | CGACTTGGGCTGCGAAAAGGCAGGGTCCGAACC | 33 |
| F1F113 | TTTCGCAGCCCAAGTCGGTGCTCGATGCGGGTC | 33 |
| F1R130 | GCACCCTGGCAGGTCAGACCCGCATCGAGCAC | 32 |
| F1F146 | TGACCTGCCAGGGTGCTTCGCCATCCTCGGTCT | 33 |
| F1R162 | GACGAGAAGGATGGGTTTGGAGACCGAGGATGGCGAA | 37 |
| F1F179 | CCAAACCCATCCTTCTCGTCCCCGGAACCGGCACCA | 36 |
| F1R199 | TCGAACGACTGTGGACCTGTGGTGCCGGTTCCGGG | 35 |
| F1F215 | CAGGTCCACAGTCGTTCGACTCGAACTGGATCCCCCT | 37 |
| F1R234 | TGTAACCCAACTGCGTTGAGAGGGGGATCCAGTTCGAG | 38 |
| F1F252 | CTCAACGCAGTTGGGTTACACACCCTGCTGGATCTCACC | 39 |
| F1R272 | GAGCATGAACGGCGGGGGTGAGATCCAGCAGGGTG | 35 |
| F1F291 | CCCGCCGTTCATGCTCAACGACACCCAGGTCAACA | 35 |
| F1R307 | GCGTTGACCATGTACTCCGTGTTGACCTGGGTGTCGTT | 38 |
| F1F326 | CGGAGTACATGGTCAACGCCATCACCGCGCTCTACG | 36 |
| F1R345 | TGTTGTTGCCCGAACCAGCGTAGAGCGCGGTGATG | 35 |
| F1F362 | CTGGTTCGGGCAACAACAAGCTTCCCGTGCTTACCT | 36 |
| F1R380 | AGACCACCCTGGGACCAGGTAAGCACGGGAAGCT | 34 |
| F1F398 | GGTCCCAGGGTGGTCTGGTTGCACAGTGGGGTC | 33 |
| F1R414 | TGATACTGGGGAAGAAGGTCAGACCCCACTGTGCAACC | 38 |
| F1F431 | TGACCTTCTTCCCCAGTATCAGGTCCAAGGTCGATCGACT | 40 |
| F1R452 | GGGCGCAAAGGCCATAAGTCGATCGACCTTGGACC | 35 |
| F1F471 | TATGGCCTTTGCGCCCGACTACAAGGGCACCGTCC | 35 |
| F1R487 | TCGAGAGGGCCGGCGAGGACGGTGCCCTTGTAGTC | 35 |
| F1F506 | TCGCCGGCCCTCTCGATGCACTCGCGGTTAGTGC | 34 |
| F1R522 | aatagGCCATACGGAGGGTGCACTAACCGCGAGTGCA | 37 |
| F1F540 | ACCCTCCGTATGGCctatttccctccgtgtgtcatga | 37 |
| F1F559 | tcatgacacacggaggga | 18 |
