# Supplementary material for: de novo Design and Synthesis of Candida antarctica Lipase B Gene and α-Factor Leads to High-Level Expression in Pichia pastoris
Source: PLoS One. 2013 Jan 10;8(1):e53939. doi: 10.1371/journal.pone.0053939 (PMC3542265; doi:10.1371/journal.pone.0053939)
Supplement: Table S3 — Oligonucleotides for the synthesis of F2 fragment of native CALB. (DOC) [file pone.0053939.s006.doc]

Table S3 Oligonucleotides for the synthesis of F2 fragment of native CALB

| ID | Sequence of oligonucleotides (5’-3’) | Number of bps |
| --- | --- | --- |
| F2R0 | TAACgagccggttgcccc | 18 |
| F2F0 | ggggcaaccggctcGTTAGTGCACCCTCCGTATGGC | 36 |
| F2R18 | CCGAACCGGTGGTTTGCTGCCATACGGAGGGTGCAC | 36 |
| F2F36 | AGCAAACCACCGGTTCGGCACTCACCACCGCACTCC | 36 |
| F2R54 | GGTCAGACCACCTGCGTTTCGGAGTGCGGTGGTGAGTG | 38 |
| F2F72 | GAAACGCAGGTGGTCTGACCCAGATCGTGCCCACCACC | 38 |
| F2R92 | CGGTCGCCGAGTAGAGGTTGGTGGTGGGCACGATCTG | 37 |
| F2F110 | AACCTCTACTCGGCGACCGACGAGATCGTTCAGCCTCAG | 39 |
| F2R129 | CGAGTGGCGAGTTGGACACCTGAGGCTGAACGATCTCGT | 39 |
| F2F149 | GTGTCCAACTCGCCACTCGACTCATCCTACCTCTTCAACGGA | 42 |
| F2R168 | CCTGTGCCTGGACGTTCTTTCCGTTGAAGAGGTAGGATGAGT | 42 |
| F2F191 | AAGAACGTCCAGGCACAGGCCGTGTGTGGGCCGCT | 35 |
| F2R210 | CCTGCATGGTCGATGACGAACAGCGGCCCACACACGG | 37 |
| F2F226 | GTTCGTCATCGACCATGCAGGCTCGCTCACCTCGCAGTT | 39 |
| F2R247 | TCGACCGACGACGTAGGAGAACTGCGAGGTGAGCGAG | 37 |
| F2F265 | CTCCTACGTCGTCGGTCGATCCGCCCTGCGCTCCA | 35 |
| F2R284 | CGAGCCTGGCCCGTGGTGGAGCGCAGGGCGGA | 32 |
| F2F300 | CCACGGGCCAGGCTCGTAGTGCAGACTATGGCATTACGG | 39 |
| F2R316 | CGGGAAGAGGGTTGCAGTCCGTAATGCCATAGTCTGCACTA | 41 |
| F2F339 | ACTGCAACCCTCTTCCCGCCAATGATCTGACTCCCGAGC | 39 |
| F2R357 | GCCGCGGCGACCTTTTGCTCGGGAGTCAGATCATTGG | 37 |
| F2F378 | AAAAGGTCGCCGCGGCTGCGCTCCTGGCGCCG | 32 |
| F2R394 | ACGATGGCTGCAGCTGCCGGCGCCAGGAGCGCA | 33 |
| F2F410 | GCAGCTGCAGCCATCGTGGCGGGTCCAAAGCAGA | 34 |
| F2R427 | AGGTCGGGCTCGCAGTTCTGCTTTGGACCCGCC | 33 |
| F2F444 | ACTGCGAGCCCGACCTCATGCCCTACGCCCGC | 32 |
| F2R460 | CCTTTTGCCTACTGCAAAGGGGCGGGCGTAGGGCATG | 37 |
| F2F476 | CCCTTTGCAGTAGGCAAAAGGACCTGCTCCGGCATCG | 37 |
| F2R497 | GCCGCTCAGGGGGTGACGATGCCGGAGCAGGT | 32 |
| F2F513 | TCACCCCCTGAGCGGCCGCtgaattccgcgccg | 33 |
| F2F529 | cggcgcggaattcaGCG | 17 |
