# Supplementary material for: de novo Design and Synthesis of Candida antarctica Lipase B Gene and α-Factor Leads to High-Level Expression in Pichia pastoris
Source: PLoS One. 2013 Jan 10;8(1):e53939. doi: 10.1371/journal.pone.0053939 (PMC3542265; doi:10.1371/journal.pone.0053939)
Supplement: Table S4 — Oligonucleotides for the synthesis of F1M fragment of codon-optimized CALB. (DOC) [file pone.0053939.s007.doc]

Table S4 Oligonucleotides for the synthesis of F1M fragment of codon-optimized CALB

| ID | Sequence of oligonucleotides (5’-3’) | Number of bps |
| --- | --- | --- |
| F1R0 | AGCTTCATaggaactccaggat | 22 |
| F1F0 | atcctggagttcctATGAAGCTGCTGTCTCTGACTGGTGT | 40 |
| F1R22 | TAGCCAAGACTCCAGCCACACCAGTCAGAGACAGC | 35 |
| F1F40 | GGCTGGAGTCTTGGCTACTTGCGTTGCAGCCAC | 33 |
| F1R57 | GCAGTCTCTTGACCAAAGGAGTGGCTGCAACGCAAG | 36 |
| F1F73 | TCCTTTGGTCAAGAGACTGCCTTCTGGTTCCGATCCAGC | 39 |
| F1R93 | GGACTTTGGCTGGGAAAAGGCTGGATCGGAACCAGAAG | 38 |
| F1F112 | CTTTTCCCAGCCAAAGTCCGTCCTGGATGCAGGTCT | 36 |
| F1R131 | AGCACCCTGGCAGGTCAGACCTGCATCCAGGAC | 33 |
| F1F148 | GACCTGCCAGGGTGCTTCCCCATCCTCTGTCTCC | 34 |
| F1R164 | GGACCAACAGGATTGGCTTGGAGACAGAGGATGGGGA | 37 |
| F1F182 | AAGCCAATCCTGTTGGTCCCAGGAACCGGAACCACT | 36 |
| F1R201 | TCGAAGGACTGTGGACCAGTGGTTCCGGTTCCTG | 34 |
| F1F218 | GGTCCACAGTCCTTCGACTCCAACTGGATTCCTCTGT | 37 |
| F1R235 | AGTGTATCCCAACTGAGTGGACAGAGGAATCCAGTTGGAG | 40 |
| F1F255 | CCACTCAGTTGGGATACACTCCATGCTGGATCTCTCCA | 38 |
| F1R275 | CGTTCAGCATGAATGGAGGTGGAGAGATCCAGCATGG | 37 |
| F1F293 | CCTCCATTCATGCTGAACGACACCCAGGTCAACACC | 36 |
| F1R312 | GATAGCGTTGACCATGTACTCGGTGTTGACCTGGGTGT | 38 |
| F1F329 | GAGTACATGGTCAACGCTATCACTGCTCTGTACGCTGG | 38 |
| F1R350 | CAGCTTGTTGTTGCCAGATCCAGCGTACAGAGCAGT | 36 |
| F1F367 | ATCTGGCAACAACAAGCTGCCTGTTCTGACTTGGTCCC | 38 |
| F1R386 | TGAGCAACCAATCCACCTTGGGACCAAGTCAGAACAGG | 38 |
| F1F405 | AAGGTGGATTGGTTGCTCAATGGGGTCTGACCTTCTT | 37 |
| F1R424 | ACCTTGGATCTGATACTTGGGAAGAAGGTCAGACCCCAT | 39 |
| F1F442 | CCCAAGTATCAGATCCAAGGTCGATCGTCTGATGGCTTTC | 40 |
| F1R463 | TACCCTTGTAGTCTGGAGCGAAAGCCATCAGACGATCG | 38 |
| F1F482 | GCTCCAGACTACAAGGGTACCccctggcctacgcc | 35 |
| F1F501 | ggcgtaggccagggGG | 16 |
