# Supplementary material for: de novo Design and Synthesis of Candida antarctica Lipase B Gene and α-Factor Leads to High-Level Expression in Pichia pastoris
Source: PLoS One. 2013 Jan 10;8(1):e53939. doi: 10.1371/journal.pone.0053939 (PMC3542265; doi:10.1371/journal.pone.0053939)
Supplement: Table S5 — Oligonucleotides for the synthesis of F2M fragment of codon-optimized CALB. (DOC) [file pone.0053939.s008.doc]

Table S5 Oligonucleotides for the synthesis of F2M fragment of codon-optimized CALB

| ID | Sequence of oligonucleotides (5’-3’) | Number of bps |
| --- | --- | --- |
| F2R0 | GGACGGTACCagaggagg | 18 |
| F2F0 | cctcctctGGTACCGTCCTGGCTGGTCCATTGGA | 34 |
| F2R18 | GCACTAACAGCCAAAGCATCCAATGGACCAGCCA | 34 |
| F2F34 | TGCTTTGGCTGTTAGTGCTCCATCTGTCTGGCAGC | 35 |
| F2R52 | AGCAGAACCAGTGGTCTGCTGCCAGACAGATGGA | 34 |
| F2F69 | AGACCACTGGTTCTGCTTTGACCACTGCTCTGCG | 34 |
| F2R86 | GTCAATCCACCAGCGTTACGCAGAGCAGTGGTCAA | 35 |
| F2F103 | TAACGCTGGTGGATTGACCCAGATCGTTCCAACTACC | 37 |
| F2R121 | CGGTAGCAGAGTACAGGTTGGTAGTTGGAACGATCTGG | 38 |
| F2F140 | AACCTGTACTCTGCTACCGACGAGATCGTTCAGCCT | 36 |
| F2R159 | TGGAGAGTTGGAGACCTGAGGCTGAACGATCTCGT | 35 |
| F2F176 | CAGGTCTCCAACTCTCCACTGGACTCCTCCTACCTG | 36 |
| F2R194 | GGACGTTCTTACCGTTGAACAGGTAGGAGGAGTCCAG | 37 |
| F2F212 | TTCAACGGTAAGAACGTCCAGGCTCAAGCTGTCTGT | 36 |
| F2R231 | GATGACGAACAGTGGACCACAGACAGCTTGAGCCT | 35 |
| F2F248 | GGTCCACTGTTCGTCATCGACCATGCTGGTTCTTTGA | 37 |
| F2R266 | ACGTAGGAGAACTGGGAAGTCAAAGAACCAGCATGGTC | 38 |
| F2F285 | CTTCCCAGTTCTCCTACGTCGTCGGTAGATCTGCTCT | 37 |
| F2R304 | CCTGTCCAGTAGTGGATCTCAGAGCAGATCTACCGACG | 38 |
| F2F322 | GAGATCCACTACTGGACAGGCTAGATCTGCTGACTACGGTA | 41 |
| F2R342 | TGGGTTACAGTCGGTGATACCGTAGTCAGCAGATCTAG | 38 |
| F2F363 | TCACCGACTGTAACCCATTGCCTGCTAACGACCT | 34 |
| F2R380 | GACCTTTTGCTCTGGAGTCAGGTCGTTAGCAGGCAA | 36 |
| F2F397 | GACTCCAGAGCAAAAGGTCGCTGCAGCTGCATTGT | 35 |
| F2R416 | GCAGCTGCTGGAGCCAACAATGCAGCTGCAGC | 32 |
| F2F432 | TGGCTCCAGCAGCTGCAGCTATCGTCGCTGGA | 32 |
| F2R448 | GCTCACAGTTCTGCTTAGGTCCAGCGACGATAGCT | 35 |
| F2F464 | CCTAAGCAGAACTGTGAGCCAGATCTGATGCCATACGC | 38 |
| F2R483 | CCAACAGCGAATGGTCTTGCGTATGGCATCAGATCTG | 37 |
| F2F502 | AAGACCATTCGCTGTTGGTAAGCGTACTTGCTCTGGTA | 38 |
| F2R520 | cgagtcaTTATGGAGTGACGATACCAGAGCAAGTACGCTTA | 41 |
| F2F540 | TCGTCACTCCATAAtgactcgcaccgcctcaagcgag | 37 |
| F2F561 | ctcgcttgaggcggtg | 16 |
