# Supplementary material for: de novo Design and Synthesis of Candida antarctica Lipase B Gene and α-Factor Leads to High-Level Expression in Pichia pastoris
Source: PLoS One. 2013 Jan 10;8(1):e53939. doi: 10.1371/journal.pone.0053939 (PMC3542265; doi:10.1371/journal.pone.0053939)
Supplement: Table S6 — Primers used in OE-PCR for amplifying the native CalBSP, CalBP and mature CALB genes. (DOC) [file pone.0053939.s009.doc]

Table S6 Primers used in OE-PCR for amplifying the native CalBSP, CalBP and mature CALB genes

| Primers | Sequences |
| --- | --- |
| *CalB*-SP | 5’- T*GGATCC*ATGAAGCTACTCTCTCTGACT -3’, *Bam*H I |
| *CalB*-P | 5’-G*GAATTC*GCCACTCCTTTGGTGAAGCGTCT-3’, *Eco*R I |
| *CalB*-F | 5’-G*GAATTC*CTACCTTCCGGTTCGGACCCT-3’, *Eco*R I |
| *CalB*-R | 5’-TATATA*GCGGCCG*CTCAGGGGGTGACGAT-3’, *Not* I |
