# Supplementary material for: de novo Design and Synthesis of Candida antarctica Lipase B Gene and α-Factor Leads to High-Level Expression in Pichia pastoris
Source: PLoS One. 2013 Jan 10;8(1):e53939. doi: 10.1371/journal.pone.0053939 (PMC3542265; doi:10.1371/journal.pone.0053939)
Supplement: Table S7 — Primers used in OE-PCR for amplifying the codon-optimized CalBSP, CalBP and mature CALB genes. (DOC) [file pone.0053939.s010.doc]

Table S7 Primers used in OE-PCR for amplifying the codon-optimized CalBSP, CalBP and mature CALB genes

| Primers | Sequences |
| --- | --- |
| *CalBM*-SP | 5’-T*GGATCC*ATGAAGCTGCTGTCTCTGACT-3’, *Bam*H I |
| *CalBM*-P | 5’-G*GAATTC*GCCACTCCTTTGGTCAAGAGACT-3’, *Eco*R I |
| *CalBM*-F | 5’-G*GAATTC*CTGCCTTCTGGTTCCGATCCA-3’, *Eco*R I |
| *CalBM*-R | 5’-G*GCGGCCGC*TTATGGAGTGACGATACCAGAGCAA-3’, *Not* I |
