# Supplementary material for: de novo Design and Synthesis of Candida antarctica Lipase B Gene and α-Factor Leads to High-Level Expression in Pichia pastoris
Source: PLoS One. 2013 Jan 10;8(1):e53939. doi: 10.1371/journal.pone.0053939 (PMC3542265; doi:10.1371/journal.pone.0053939)
Supplement: Table S8 — Codon usage frequency of amino acid of original and optimized CALB gene in Pichia pastoris. (DOC) [file pone.0053939.s011.doc]

Table S8 Codon usage frequency of amino acid of original and optimized CALB gene in *Pichia pastoris*.

| Amino Acids | Codon | Frequency in *Pichia* genome  (/1000) | Frequency in native CALB gene  (/1000) | Frequency in codon-optimized CALB gene  (/1000) |
| --- | --- | --- | --- | --- |
| Alanine, A | GCT | 29.9 | 17.5 | 90.6 |
|  | GCC | 17.0 | 32.1 | 5.8 |
|  | GCA | 16.4 | 37.9 | 20.5 |
|  | GCG | 3.5 | 29.2 | 0.0 |
| Cysteine, C | TGT | 8.3 | 2.9 | 8.8 |
|  | TGC | 4.4 | 17.5 | 11.7 |
| Aspartate, A | GAT | 36.8 | 11.7 | 14.6 |
|  | GAC | 26.5 | 29.2 | 26.3 |
| Glycine, G | GGT | 27.1 | 46.6 | 52.6 |
|  | GGA | 20.0 | 5.8 | 26.3 |
|  | GGC | 8.5 | 26.2 | 2.9 |
| Histidine, H | CAT | 10.4 | 2.9 | 2.9 |
| Lysine, K | AAG | 34.8 | 26.2 | 32.2 |
|  | AAA | 30.0 | 5.8 | 0.0 |
| Leucine, L | TTG | 31.9 | 5.8 | 38.0 |
|  | CTG | 15.5 | 26.2 | 67.3 |
|  | CTT | 16.1 | 17.5 | 0.0 |
|  | CTC | 7.6 | 49.6 | 0.0 |
| Methionine, M | ATG | 19.2 | 14.6 | 14.6 |
| Asparagine, N | AAC | 25.5 | 37.9 | 40.9 |
|  | AAT | 22.9 | 2.9 | 0.0 |
| Proline, P | CCA | 17.5 | 11.7 | 67.3 |
|  | CCT | 15.3 | 17.5 | 23.4 |
|  | CCC | 6.5 | 49.6 | 0.0 |
| Glutanine, Q | CAA | 24.1 | 5.8 | 11.7 |
|  | CAG | 14.5 | 46.6 | 40.9 |
| Arginine, R | AGA | 19.9 | 0.0 | 17.5 |
|  | CGT | 6.7 | 5.8 | 8.8 |
|  | AGG | 6.7 | 5.8 | 0.0 |
| Serine, S | TCT | 23.9 | 2.9 | 38.0 |
|  | TCC | 16.3 | 35.0 | 49.7 |
|  | TCG | 6.8 | 37.9 | 0.0 |
| Threonine, T | ACT | 23.7 | 8.7 | 49.7 |
|  | ACC | 14.0 | 61.2 | 38.0 |
|  | ACA | 14.2 | 5.8 | 0.0 |
|  | ACG | 6.0 | 11.7 | 0.0 |
| Valine, V | GTT | 27.0 | 11.7 | 20.5 |
|  | GTC | 14.7 | 35.0 | 55.6 |
|  | GTG | 12.8 | 26.2 | 2.92 |
|  | GTA | 10.0 | 5.8 | 0.0 |
| Tyrosine, Y | TAC | 18.3 | 23.3 | 26.3 |
